# 

**Published:** 1885-08

**Authors:** 


### WANTED.

A young man (without family preferred), entirely competent to do first class work, and *reliable*, to take the place of an established practitioner who will be absent from his office from September 1st until spring.

No one who cannot fully answer the requirements need apply.

Address,

"M. D."

Care of the Editor of the INDEPENDENT PRACTITIONER,

Buffalo, N. Y.

